# SLC2A5 Correlated with Immune Infiltration: A Candidate Diagnostic and Prognostic Biomarker for Lung Adenocarcinoma

**DOI:** 10.1155/2021/9938397

**Published:** 2021-09-23

**Authors:** Lianxiang Luo, Jiating Su, Yushi Zheng, Fangfang Huang, Riming Huang, Hui Luo

**Affiliations:** ^1^Southern Marine Science and Engineering Guangdong Laboratory (Zhanjiang), Zhanjiang, Guangdong 524023, China; ^2^The Marine Biomedical Research Institute, Guangdong Medical University, Zhanjiang, Guangdong 524023, China; ^3^The Marine Biomedical Research Institute of Guangdong Zhanjiang, Zhanjiang, Guangdong 524023, China; ^4^The First Clinical College, Guangdong Medical University, Zhanjiang, Guangdong 524023, China; ^5^Graduate School, Guangdong Medical University, Zhanjiang, Guangdong 524023, China; ^6^Guangdong Provincial Key Laboratory of Food Quality and Safety, College of Food Science, South China Agricultural University, Guangzhou, China

## Abstract

Lung adenocarcinoma (LUAD) is a major subtype of lung cancer with a relatively poor prognosis, requiring novel therapeutic approaches. Great advances in new immunotherapy strategies have shown encouraging results in lung cancer patients. This study is aimed at elucidating the function of SLC2A5 in the prognosis and pathogenesis of LUAD by analyzing public databases. The differential expression of SLC2A5 in various tissues from Oncomine, GEPIA, and other databases was obtained, and SLC2A5 expression at the protein level in normal and tumor tissues was detected with the use of the HPA database. Then, we used the UALCAN database to analyze the expression of SLC2A5 in different clinical feature subgroups. Notably, in both PrognoScan and Kaplan-Meier plotter databases, we found a certain association between SLC2A5 and poor OS outcomes in LUAD patients. Studies based on the TIMER database show a strong correlation between SLC2A5 expression and various immune cell infiltrates and markers. The data analysis in the UALCAN database showed that the decreased promoter methylation level of SLC2A5 in LUAD may lead to the high expression of SLC2A5. Finally, we used the LinkedOmics database to evaluate the SLC2A5-related coexpression and functional networks in LUAD and to investigate their role in tumor immunity. These findings suggest that SLC2A5 correlated with immune infiltration can be used as a candidate diagnostic and prognostic biomarker in LUAD patients.

## 1. Introduction

Lung cancer is considered to be one of the most common cancers worldwide with high incidence rate and high mortality rate [1]. Non-small-cell lung cancer, including lung adenocarcinoma (LUAD) and lung squamous cell carcinoma, is the main type of lung cancer, accounting for nearly 85%; among them, LUAD is the most common histological subtype [2]. LUAD generally originates from the surrounding lung tissue [3] and is characterized by the formation of glands or ducts and the production of a large amount of mucus, with obvious cellular and molecular characteristics [4]. Due to the high metastasis, recurrence, and drug resistance of LUAD, the curative effect of conventional treatment including surgery, radiotherapy, and chemotherapy is not so satisfactory, and the incidence of this aggressive disease is still surprisingly high [5, 6]. Therefore, it is urgent to find reliable biomarkers for the diagnosis and prognosis prediction of LUAD, which will contribute to the effective treatment of LUAD.

SLC2A5, namely, fructose transporter GLUT5 gene, is one of the key genes in the process of tumor development, whose regulation mechanism is the regulation of fructose uptake and absorption and carbon absorption in cells [7]. Studies have found that the expression of SLC2A5 is closely related to the metabolism of tumor cells and tumor progression, especially the increase of SLC2A5 coding GLUT5 protein seems to be associated with the development and metastasis of LUAD [7]. According to recent reports, SLC2A5 is involved in the progression of a variety of cancers, including pancreatic cancer, breast cancer, small intestine carcinoma, and LUAD [7]. SLC2A5 can increase the flux of pentose phosphate pathway and protein synthesis by increasing fructose synthesis, thus promoting the growth of pancreatic cancer [8]. Jiang et al. [9] demonstrated that SLC2A5 can increase the risk of breast cancer development and metastasis by promoting fructose synthesis, which could induce lipoxygenase-12 and related fatty acid 12-HETE in breast cancer cells [9]. A study on acute myeloid leukemia (AML) showed that SLC2A5 upregulation occurs in AML, and blocking the pharmacological process of SLC2A5 regulating fructose uptake could improve the phenotype of leukemia [10]. Moreover, studies have shown that SLC2A5 are significantly upregulated in LUAD patients and that their overexpression is highly correlated with poor survival in LUAD patients [7]. However, whether SLC2A5 is a powerful biomarker of LUAD has not been given a clear answer. Also, the biological functions of SLC2A5 in LUAD remain to be determined.

As is known to us, inflammation is one of the major contributors to the tumor microenvironment [11], and the risk of cancer is greatly increased by viral and bacterial infection [12]. Previous studies have found increases in the expression of indoleamine 2,3-dioxygenase 1 (IDO1) associated with intestinal flora and the differentiation of gut secretion cells is related to inflammation, injury, infection, changes in flora, and so on, but with reduced SLC2A5 levels [13]. This may indicate that changes in SLC2A5 levels are associated with inflammation, viruses, flora changes, infection, etc., thus affecting tumor occurrence and/or progression. The above thinking may provide promising foundations and analysis for studying the role that SLC2A5 plays in lung cancer.

To better explore the role of SLC2A5 in LUAD, we learned the research ideas of Luo et al. in this study [14]. We first explored the differential expression of SLC2A5 in different organizations using databases like Oncomine and GEPIA. Meanwhile, the Human Protein Atlas (HPA) database was used to detect SLC2A5 expression in normal and tumor tissues. Moreover, we used the UALCAN database to analyze the expression of SLC2A5 in different clinical feature subgroups. PrognoScan database and Kaplan-Meier plotter were used to assess the relationship between SLC2A5 and prognosis of LUAD patients comprehensively. Tumor immunoassay resource (TIMER) database was used to further investigate the association between SLC2A5 expression levels and immune cell infiltration, different immune cell subsets markers in LUAD, and the UALCAN database was used to analyze the promoter methylation level of SLC2A5 in LUAD and the correlation between SLC2A5 expression level and different subgroups of LUAD patients. Finally, we used the LinkedOmics database to evaluate the SLC2A5-related coexpression and functional network of SLC2A5 in LUAD and to investigate their role in tumor immunity. Our results provide a novel insight into the function of SLC2A5 in LUAD and a theoretical basis for the early diagnosis, prognosis, and targeted therapy of LUAD.

## 2. Materials and Methods

### 2.1. Oncomine Database Analysis

The web-based Oncomine database, whose data includes a microarray database of most human cancers, aims at facilitating cancer-related factor discovery from genome-wide expression analyses [15, 16]. In this study, following the methods of Ma et al. [17], we conducted Oncomine database analysis to assess SLC2A5 expression level based on the following criteria: (1) “gene: SLC2A5”; (2) “analysis type: cancer vs. normal”; (3) “cancer type: lung cancer”; (4) “data type: mRNA”; and (5) threshold settings: folding change = 2, *P* value = 0.05.

### 2.2. TIMER Database Analysis

By studying the research ideas of Luo et al. [14], the TIMER database is an effective tool to analyze the abundance of tumor-infiltrating immune cells from the target gene expression data [18–20]. Thus, this study analyzed the expression of SLC2A5 in LUAD patients and six types of infiltration of immune cells (B cells, CD4+ T cells, CD8+ T cells, neutrophils, macrophages, and dendritic cells). At the same time, the correlation between SLC2A5 expression and the genetic markers of tumor-infiltrating immune cells was also discussed.

### 2.3. GEPIA Database Analysis

The GEPIA (Gene Expression Profiling Interactive Analysis) web server has been a valuable and highly cited resource for gene expression analysis based on tumor and normal samples from TCGA and the GTEx databases [21–23]. So the expression of SLC2A5 in tumor and normal tissues of this study was further used to conduct with GEPIA, as shown in the box diagram.

### 2.4. UALCAN Database Analysis

In this study, UALCAN, an interactive web portal based on TCGA3RNA-seq and clinical data of 31 cancer types [24], was used to analyze the correlation between SLC2A5 expression and different clinical factors.

### 2.5. Kaplan-Meier Plotter Analysis

The Kaplan-Meier plotter is an online database including gene expression data and clinical data, which offers a means of exploring the impact of a wide variety of genes on patient survival in 21 different types of cancer [25, 26]. Therefore, this database was used to evaluate the prognostic value of SLC2A5 and explore the association between SLC2A5 expression and outcome in LUAD patients, as well as the impact of both clinicopathological factors and SLC2A5 on the outcome of patients with LUAD.

### 2.6. Human Protein Atlas Database Analysis

The Human Protein Atlas (HPA) [27, 28] provides a powerful platform to evaluate protein localization and expression in human cells, tissues, and organs [29]. Therefore, we used the database to analyze the protein expression and immunohistochemistry (IHC) of SLC2A5 in normal and LUAD tissues.

### 2.7. PrognoScan Database Analysis

The PrognoScan database offers a valuable tool for researchers assessing the biological relationship between gene expression and prognosis, which is of great help to accelerate our cancer research [30, 31]. This database was used to assess the relationship between SLC2A5 expression and patient outcome.

### 2.8. Meta-Analysis

Meta-analysis can evaluate the evidence and the effect indicators more accurately and objectively, so as to explain the heterogeneity of different research results [32, 33]. Therefore, meta-analysis was used to evaluate the overall prognostic significance of SLC2A5 in LUAD patients. HR and 95% CI were calculated to evaluate the correlation between SLC2A5 expression and the prognosis of LUAD patients. With the random effects model, the heterogeneity across multiple datasets was assessed by the *Q* test (*I*^2^ statistics).

### 2.9. LinkedOmics Database Analysis

The LinkedOmics database contains multiomic cancer datasets for 32 cancer types and a total of 11,158 patients from TCGA project. This comprehensive and functional database also provides three key analysis modules including LinkFinder, LinkCompare, and LinkInterpreter [34] and an analysis toolkit (WebGestalt) [35, 36], which were applied in this study. Thus, LinkedOmics was used to sign and rank the data which was selected for GSEA to perform GO (BP, CC, and MF) and KEGG analysis in this study.

## 3. Results

### 3.1. High SLC2A5 Expression in LUAD

**Figure 1:**
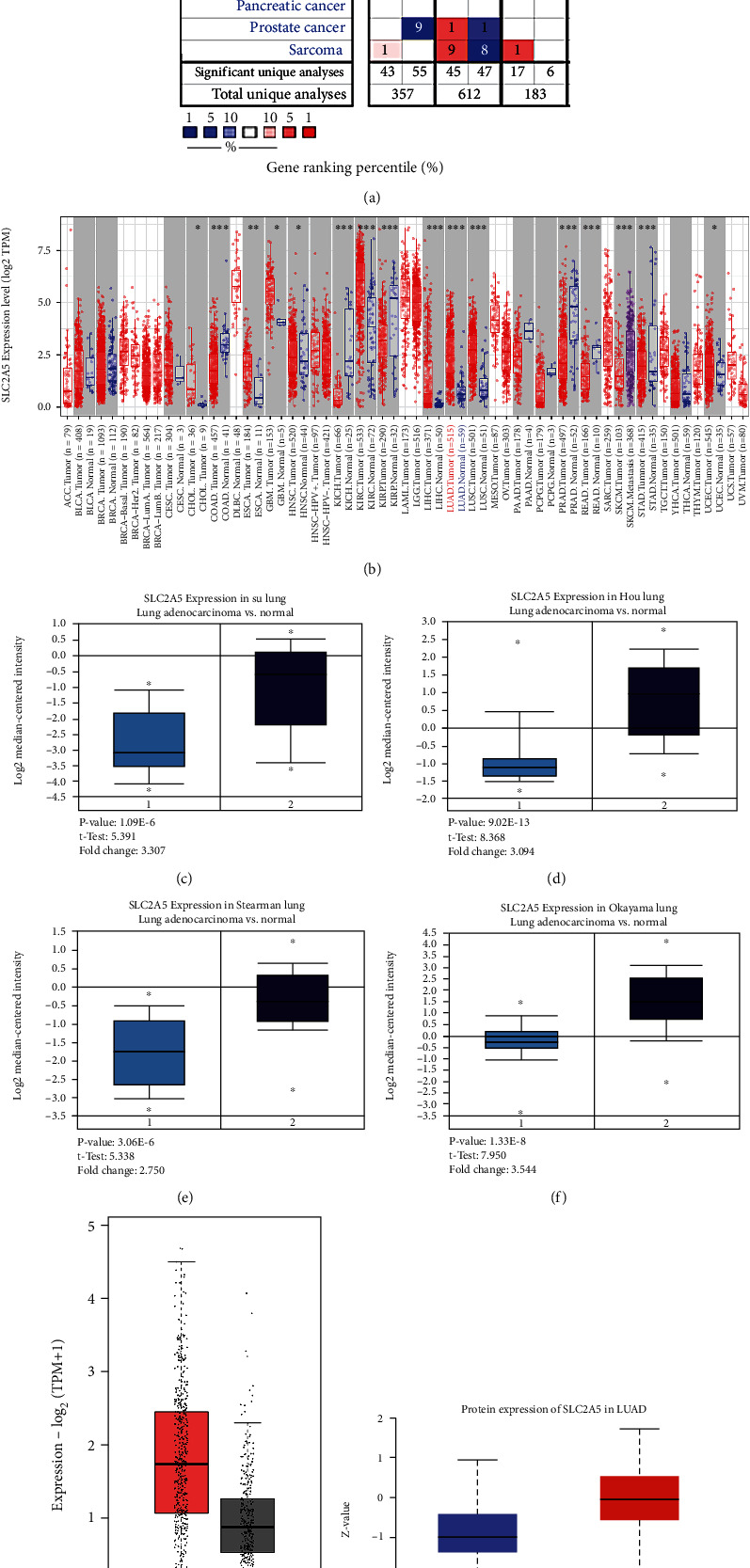
SLC2A5 is highly expressed in LUAD. (a) The expression level of SLC2A5 in different types of tumor tissues and normal tissues in the Oncomine database. (*P* value is 0.05, data type is mRNA, and gene ranking of all.) (b) The expression level of SLC2A5 in different types of tumor tissues and normal tissues in the TIMER database (^∗^*P* < 0.05, ^∗∗^*P* < 0.01, and ^∗∗∗^*P* < 0.001). (c–f) The expression of SLC2A5 in LUAD is higher than that in normal tissues in different databases (SLC2A5 expression in Su's dataset, Hou's dataset, Stearman's dataset, and Okayama's dataset) in Oncomine. (g) High expression of SLC2A5 mRNA in LUAD tissues (*n* = 483) compared with the normal tissues (*n* = 347) in the GEPIA database. (h) High protein expression of SLC2A5 in LUAD tissues (*n* = 111) compared with the normal tissues (*n* = 111) in the UALCAN database. (i) Immunohistochemistry (IHC) of SLC2A5 expression in LUAD tissues and corresponding normal tissues based on HPA.

Oncomine and TIMER databases were used to evaluate the mRNA expression of SLC2A5 in multiple tumor tissues and normal tissues. Oncomine analysis results showed that in several solid tumors, the expression of SLC2A5 was more significant in lung cancer, lymphatic cancer, brain and CNS cancer, and breast cancer, while the expression rate of SLC2A5 in other cancers such as gastric cancer, ovarian cancer, and other cancers was lower (Figure 1(a)). Meanwhile, Oncomine analysis of LUAD and normal samples also showed that in different patient datasets (SLC2A5 expression in Su's dataset, Hou's dataset, Stearman's dataset, and Okayama's dataset), the expression of SLC2A5 in LUAD was higher than that in normal lung gland tissue (Figures 1(c)–1(f)). TIMER database analysis results revealed the SLC2A5 expression differences in all tumor tissues and adjacent normal tissues (Figure 1(b)): SLC2A5 expression in COAD (colon adenocarcinoma), KICH (renal cell carcinoma), KIRP (renal cell carcinoma), PRAD (prostate adenocarcinoma), and READ (rectal adenocarcinoma) was significantly lower than that in adjacent normal tissues; in contrast, SLC2A5 expression in LUAD (lung adenocarcinoma), UCEC (endometrial carcinoma), CHOL (cholangiocarcinoma), KIRC (renal clear cell carcinoma), LIHC (hepatocellular carcinoma), LUSC (lung squamous cell carcinoma), and ESCA (esophageal carcinoma) was significantly higher. Data mining of GEPIA and UALCAN databases was further confirmed. On the one hand, GEPIA analysis confirmed that the expression of SLC2A5 in LUAD tissues was significantly upregulated compared with normal lung gland tissues (*P* < 0.05) (Figure 1(g)). On another, analysis in UALCAN examined the protein expression of SLC2A5 and found it was highly expressed in tumor tissues (Figure 1(h)). In addition, we also carried out immunohistochemistry (IHC) in the HPA database. As can be seen from Figure 1(i), the protein expression of SLC2A5 in normal tissues decreased significantly, while the protein level in tumor tissues increased significantly. According to the results of differential analysis of SLC2A5 above, SLC2A5 is highly expressed in LUAD, suggesting that abnormal expression of SLC2A5 may be closely related to the development, metastasis, and prognosis of LUAD.

### 3.2. Distribution of SLC2A5 Expression in Clinical Characteristic Subgroups

**Figure 2:**
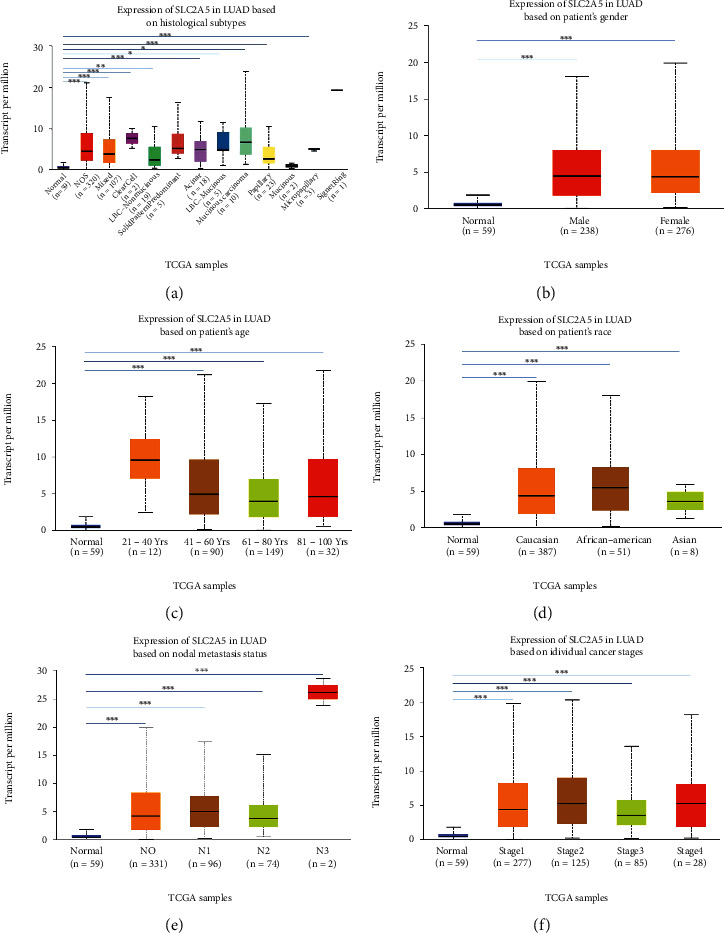
SLC2A5 expression in subgroups of clinical characteristics. (a) Expression of SLC2A5 in LUAD based on histological subtypes. (b) Expression of SLC2A5 in LUAD based on patient's gender. (c) Expression of SLC2A5 in LUAD based on patient's age. (d) Expression of SLC2A5 in LUAD based on patient's race. (e) Expression of SLC2A5 in LUAD based on nodal metastasis status. (f) Expression of SLC2A5 in LUAD based on individual cancer stages.

Using the UALCAN database to detect the distribution of SLC2A5 in different histological subtypes of LUAD, it was found that the expression of SLC2A5 in lung adenocarcinoma-not otherwise specified (NOS), lung adenocarcinoma mixed subtype (Mixed), lung clear cell adenocarcinoma (ClearCell), lung bronchioloalveolar carcinoma nonmucinous (LBC-Nonmucinous), lung solid pattern predominant adenocarcinoma (SolidPatternPredominant), lung acinar adenocarcinoma (Acinar), lung bronchioloalveolar carcinoma mucinous (LBC-Mucinous), mucinous (colloid) carcinoma (Mucinous), lung papillary adenocarcinoma (Papillary), lung mucinous adenocarcinoma (Mucinous), lung micropapillary adenocarcinoma (Micropapillary), and lung signet ring adenocarcinoma (SignetRing) was significantly higher than that of normal LUAD (Figure 2(a)). Subgroup analysis based on sex, age, race, different lymph node metastasis status, and tumor stage showed that the expression level of SLC2A5 in LUAD patients increased relative to normal samples (Figures 2(b)–2(f)). At the same time, UALCAN analysis showed that there was statistical difference between SLC2A5 expression and each tumor stage (*P* < 0.05). And the distribution of SLC2A5 increased in all stages of tumor; however, the expression of SLC2A5 did not increase with the increase of tumor stage, that is to say, there was no linear relationship between the expression of SLC2A5 and tumor stage (Figure 2(f)). Moreover, as shown in Figure 2(e), SLC2A5 expression increased in LUAD patients with different lymph node metastasis status (N classification). Nevertheless, there is no significant difference in the distribution of SLC2A5 in N0, N1, and N2 classification, and it was highly expressed in N3. Notably, SLC2A5 expression is increased in different genders of LUAD patients, and different genders were also associated with SLC2A5 expression.

### 3.3. Prognosis Analysis of SLC2A5 Expression and LUAD and Meta-Analysis of SLC2A5 Overexpression and OS of LUAD

**Figure 3:**
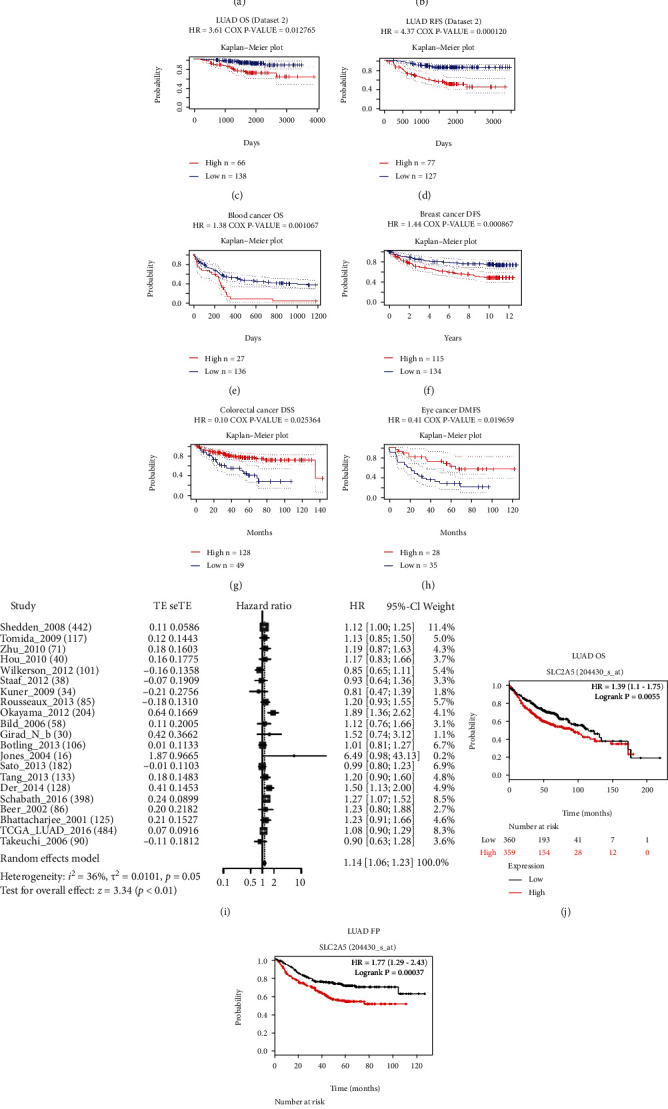
SLC2A5 is associated with survival outcome. (a–d) There was a significant correlation between prognosis in LUAD patients and expression of SLC2A5. (e–h) Several other types of cancer show a correlation between the patient's prognostic period and the expression of SLC2A5. (i) Forest plot of survival analysis results associated with high SLC2A5 expression. (j, k) Survival analyses of SLC2A5 by the Kaplan-Meier plotter web tool. (j) Overall survival (OS) of LUAD (*P* < 0.5) on SLC2A5 gene expression. (k) First progression (FP) of LUAD (*P* < 0.5) on SLC2A5 gene expression. Survival differences are compared between patients with high and low (grouped according to median) expression of SLC2A5. The numbers below the figures denote the number of patients at risk in each group.

Next, we used the PrognoScan database to explore the relationship between SLC2A5 expression and the prognosis of LUAD patients. It is found that there was a significant correlation between the prognosis of LUAD patients and the expression of SLC2A5 (*P* < 0.05) (Figures 3(a)–3(d)). Interestingly, there are many other cancer types showing certain correlation between prognosis and SLC2A5 expression, including blood cancer, breast cancer, colorectal cancer, and eye cancer (Figures 3(e)–3(h)). To strengthen the persuasive power of association between SLC2A5 high expression and poor survival analysis results of LUAD patients, we conducted a meta-analysis of different datasets. Meta-analysis results have shown that the combined HR and 95% CI of survival analysis results associated with high SLC2A5 expression were 1.14 (1.06, 1.23), and no significant heterogeneity was observed in the 21 datasets (*I*^2^ = 36%, *P* = 0.05) (Figure 3(i)). Therefore, through the meta-analysis, we can safely conclude that high expression of SLC2A5 is a powerful predictor of adverse prognosis in LUAD patients.

Kaplan-Meier plotter was further used to validate SLC2A5 high expression prognosis. According to the median level of SLC2A5 expression in each group, the patients were divided into two groups. It was found that the increased expression of SLC2A5 was associated with the prognosis of LUAD patients (*P* < 0.05), and overall survival and first progression (FP) were also highly affected by the increased expression of SLC2A5 mRNA (OS: HR = 1.39, log-rank *P* = 0.0055; FP: HR = 1.77, log-rank *P* = 0.00037) (Figures 3(j) and 3(k)), suggesting that SLC2A5 may be a reliable biomarker for the prognosis of LUAD. Since SLC2A5 expression has an impact on the prognosis of LUAD patients, we next evaluated the correlation between SLC2A5 expression level and different clinical features of LUAD by using the Kaplan-Meier plotter database to explore its potential mechanism. The results are shown in Table 1, which showed that high SLC2A5 expression did not correlate with OS and FP in female (OS: HR = 1.15, *P* = 0.4656; FP: HR = 1.35, *P* = 0.199), stage 2 (OS: HR = 0.75, *P* = 0.2407; FP: HR = 1.15, *P* = 0.6103), AJCC stage T2 (OS: HR = 0.93, *P* = 0.7933; FP: HR = 0.86, *P* = 0.6515), AJCC stage N0 (OS: HR = 1.5, *P* = 0.097; FP: HR = 1.63, *P* = 0.2142), and AJCC stage N1 (OS: HR = 0.73, *P* = 0.4358; FP: HR = 1.06, *P* = 0.8979). However, in male (OS: HR = 1.46, *P* = 0.0232; FP: HR = 1.94, *P* = 0.0028), stage 1 (OS: HR = 1.62, *P* = 0.0156; FP: HR = 2.33, *P* = 0.0007), and exclude those never smoked (OS: HR = 2.68, *P* = 6.7*e* − 05; FP: HR = 2.05, *P* = 0.0013), high SLC2A5 mRNA expression correlated with both OS and FP. In conclusion, it is proved that most other factors can independently predict the prognosis of LUAD. Therefore, SLC2A5 may be a potential independent risk factor in LUAD.

### 3.4. Correlation between SLC2A5 Expression and Immune Cell Infiltration in LUAD

**Figure 4:**
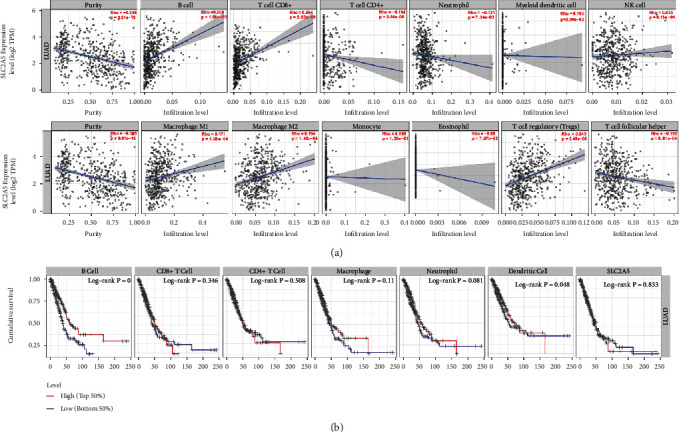
Correlation analysis between SLC2A5 expression and the infiltrating immune cells in LUAD. (a) Correlation of SLC2A5 expression with 12 types of immune infiltration cells obtained from TIMER (purity-corrected Spearman test). (b) Overall survival curve of the six types of cells produced by Kaplan-Meier estimator from TIMER. Survival differences are compared between patients with high and low (grouped according to median) infiltration of each kind of immune cells.

The TIMER database was used to analyze whether SLC2A5 high expression in LUAD tissues was associated with several major infiltrating immune cells. As the analysis results showed, the SLC2A5 expression level is correlated with B cell (Rho = 0.268, *P* = 1.59*e* − 09), CD8 T cell (Rho = 0.264, *P* = 2.63*e* − 09), CD4 T cell (Rho = −0.184, *P* = 3.84*e* − 05), neutrophil (Rho = −0.121, *P* = 7.34*e* − 03), dendritic cell (Rho = 0.105, *P* = 2.00*e* − 02), macrophage M1 (Rho = 0.171, *P* = 1.38*e* − 04), macrophage M2 (Rho = 0.194, *P* = 1.40*e* − 05), Tregs (Rho = 0.245, *P* = 3.45*e* − 08), and T cell follicular helper (Tfh) (Rho = −0.152, *P* = 6.81*e* − 04). As shown in the scatter plot (Figure 4(a)), SLC2A5 expression level was positively correlated with immune cells including B cell, CD8 T cell, Tregs, macrophage M2, macrophage M1, and DC cell and was negatively correlated with immune cells like neutrophil, Tfh, and CD8 T cell, suggesting that SLC2A5 expression level was closely correlated with LUAD immune infiltration. To further investigate the relationship between immune cell infiltration and SLC2A5 expression in LUAD, we further conducted Kaplan-Meier maps by using the TIMER database to evaluate the prognostic value of each of the six types of cells of the immune cells mentioned above (Figure 4(b)). We found that the expression of B cells (log-rank *P* = 0) and dendritic cells (log-rank *P* = 0.048) was significantly correlated with the prognosis of LUAD, which indicated that SLC2A5 plays an important role in regulating the infiltration of immune cells in LUAD, especially in the infiltration of B cells and dendritic cells.

### 3.5. Correlation Analysis between SLC2A5 mRNA Levels and Different Subgroup Markers of Immune Cells

Next, based on the set of immunologic markers in LUAD, the TIMER database was used to further search the association between SLC2A5 expression and immune cell infiltration level. Specifically, targeting the special cell subsets (including CD8+ cells, T cells (general), B cells, monocytes, TAM, M1 macrophages, M2 macrophages, neutrophils, natural killer cells, and democratic cells), we evaluated the correlation between SLC2A5 expression and levels of parkers. At the same time, we also analyzed different subsets of T cells, namely, T helper 1 (Th1), T helper 2 (Th2), follicular helper T (TFH), T helper 17 (Th17), regulatory T (Tregs), and T cell exhaustion. Since the tumor purity of clinical samples affects the immune osmotic analysis, we adjusted the results according to the tumor purity. The results showed that the expression of SLC2A5 in LUAD tissues was significantly related to the expression of most marker genes in immune osmotic cells (Table 2).

It was found that the expression of SLC2A5 in LUAD was significantly correlated with the expression of immune marker genes in CD8+ T cells, T cells, B cells, monocytes, TAM, and M2 macrophages. In particular, CD8A and CD8B of CD8+ T cells; CD3D, CD3E, and CD2 of T cells; CD19 and CD79A of B cells; CSF1R of monocytes; CCL2, CD68, and IL10 of TAMs; IRF5 of M1 macrophage; and CD163, VSIG4, and MS4A4A of M2 macrophage were all closely related to SLC2A5 level in LUAD (*P* < 0.0001). Moreover, high expression of SLC2A5 is associated with dense natural killer cell infiltration in LUAD. The expression of marker genes of natural killer cells (KIR2DL3, KIR2DL4, KIR3DL2, KIR3DL3, and KIR2DS4) was all significantly correlated with SLC2A5 expression, which suggests a close relationship between SLC2A5 expression and natural killer cell infiltration. In addition, for neutrophils, SLC2A5 expression was positively correlated with ITGAM and negatively correlated with CEACAM8. We also found that SLC2A5 expression is closely associated with TH1, Treg, and T cell failure marker genes (e.g., TBX21, STAT4, STAT1, IFNG, TNF, FOXP3, CCR8, STAT5B, TGFB1, PDCD1, LAG3, havcr2, and GZMB), which further supports the close relationship between SLC2A5 expression and LUAD immune infiltration.

### 3.6. Analysis of SLC2A5 Promoter Methylation Levels in LUAD

**Figure 5:**
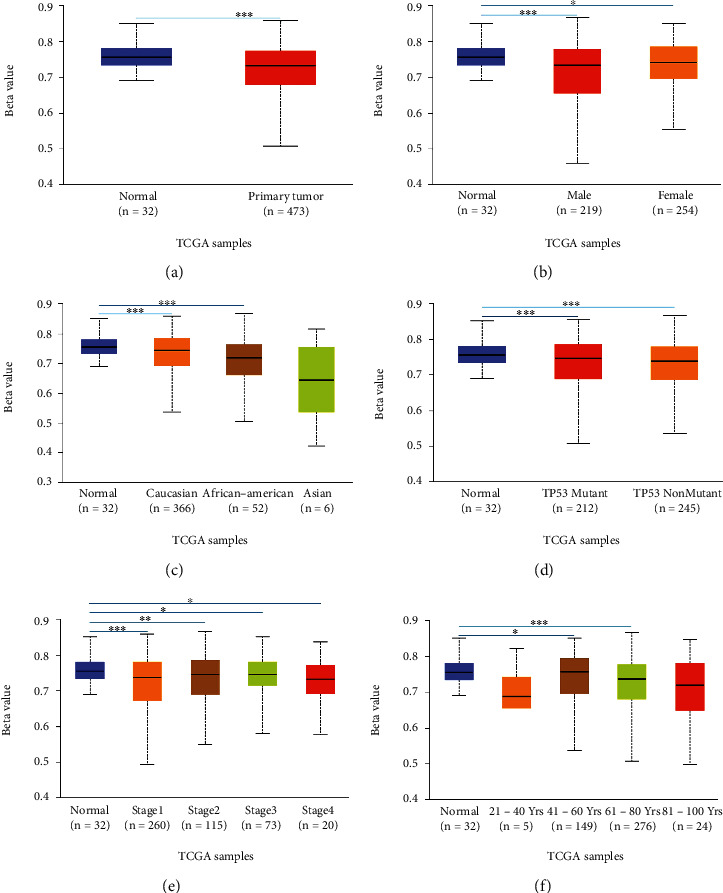
Promoter methylation levels of SLC2A5 in LUAD. Promoter methylation levels of SLC2A5 were low in (a)–(f). LUAD: (a) sample type, (b) gender, (c) race, (d) TP53 mutation status, (e) individual cancer stages, and (f) age (^∗^*P* < 0.05, ^∗∗^*P* < 0.01, and ^∗∗∗^*P* < 0.001).

A significant increase in SLC2A5 expression was found in LUAD through the above analysis. Consequently, we would conduct further studies to explore the causes of SLC2A5 elevation. Methylation is a significant event in epigenetic modification of the genome, and particularly, low global methylation can result in genomic instability and changes in gene transcription which may have an impact on normal cell growth and increase the likelihood of tumorigenesis [37]. Therefore, we used the UALCAN database to verify the methylation level of SLC2A5 promoter in LUAD. As shown in Figure 5, the promoter methylation level of SLC2A5 in normal tissues is slightly lower than that in LUAD. Moreover, we conducted subgroup analyses of promoter methylation based on the patient's race, age, sex, tumor stage, and TP53 mutation status. The results showed that the promoter methylation was associated with gender, tumor stage, and TP53 mutation status in LUAD patients (Figures 5(b), 5(d), and 5(e)); however, there was no correlation between promoter methylation and LUAD patients who aged 21-40 or 81-100 and patients of African ethnicity (Figures 5(c) and 5(f)). These findings suggest that lower promoter DNA methylation may lead to high expression levels of SLC2A5 in LUAD.

### 3.7. Gene Enrichment Analysis and Gene Coexpression Network Construction

**Figure 6:**
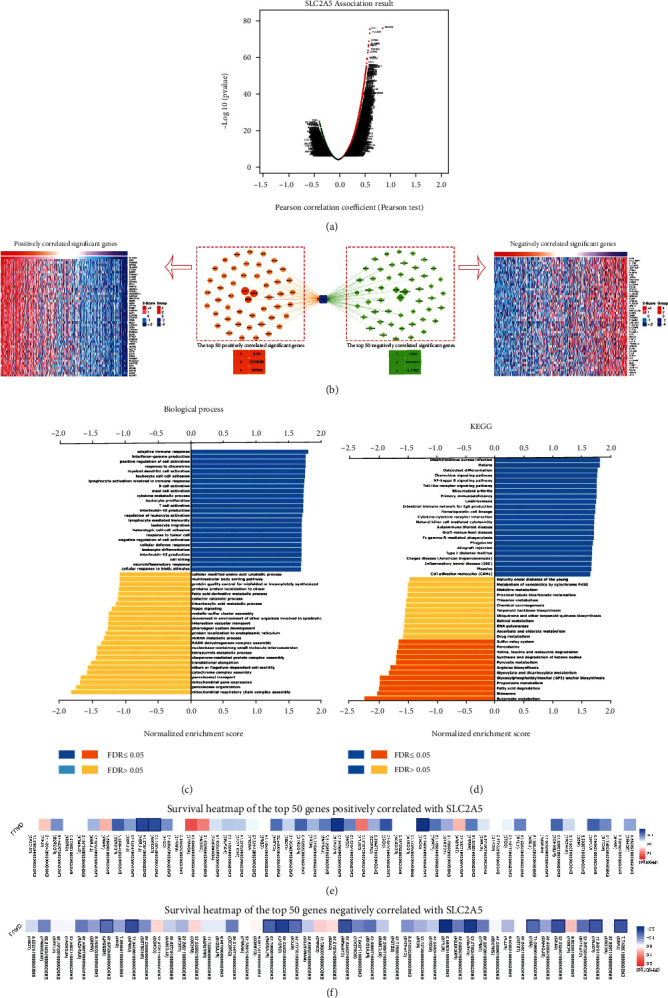
SLC2A5 coexpression genes in LUAD (LinkedOmics database). (a) The global SLC2A5 highly correlated genes were identified by the Pearson test in LUAD. Red and green dots represent positively and negatively significantly correlated genes with SLC2A5, respectively. (b) Heat maps and network showing the top 50 genes positively and negatively correlated with SLC2A5 in LUAD. (c, d) Significantly enriched GO: biological process annotations and KEGG pathways of SLC2A5 in LUAD. (e, f) Survival heat maps of the top 50 genes positively and negatively correlated with SLC2A5 in LUAD. The survival heat maps show the hazard ratios in the logarithmic scale (log10) for different genes. The red and blue blocks denote higher and lower risks, respectively. The rectangles with frames mean the significant unfavorable and favorable results in prognostic analyses (*P* < 0.05).

To better understand the biological implication of SLC2A5 in LUAD, the “LinkFinder” module in LinkedOmics database was used to examine the SLC2A5 coexpression pattern. As shown in Figure 6(a), it is indicated that 11,085 genes (red dots) were positively correlated with SLC2A5, while 8903 genes (green dots) were negatively correlated (*P* < 0.05). As shown in Figure 6(b), the first 50 positive and negative genes associated with SLC2A5 are shown in the form of heat map, and the network of them is also shown. SLC2A5 expression is strongly positively correlated with the expression of genes such as IL4I1 (positive rank #1, *r* = 0.716, *P* = 3.59*E* − 82), FCGR2B (positive rank #2, *r* = 0.708, *P* = 1.45*E* − 79), and GPR84 (positive rank #3, *r* = 0.692, *P* = 1.49*E* − 74), but negatively correlated with the expression of genes such as TOB1 (negative rank #1, *r* = −0.430, *P* = 1.40*E* − 24), SELENBP1 (negative rank #2, *r* = −0.420, *P* = 2.15*E* − 23), and IL17RE (negative rank #3, *r* = −0.416, *P* = 6.06*E* − 23). The annotation of gene set enrichment analysis (GSEA) indicates that SLC2A5-coexpressed genes are mainly involved in adaptive immune response, interferon-gamma production, positive regulation of cell activation, response to chemokine, myeloid dendritic cell activation, and other biological processes (Figure 6(c)). KEGG analysis results show that the genes are mainly enriched in *Staphylococcus aureus* infection, malaria, osteoclast differentiation, chemokine signaling pathway, NF-kappa B signaling pathway, and other pathways. In contrast, genes enriched in butanoate metabolism, ribosome, fatty acid degradation, propanoate metabolism, glycosylphosphatidylinositol (GPI) anchor biosynthesis, or others were inhibited (Figure 6(d)). Besides, we plotted the survival heat map of the genes significantly associated with SLC2A5 expression in Figures 6(e) and 6(f). As shown in the figure, only 4 of the first 50 positively related genes have lower risk ratios (HR), compared with 12/50 negative related genes which have low HR (*P* < 0.05). And all of these genes have a high probability of becoming risk ratio markers of LUAD.

## 4. Discussion

Lung cancer (LC) is a major health problem and one of the most common causes of tumor-related deaths. Its rapid growth requires excessive catabolism of major metabolic fuels [38]. In the presence of metabolic fuels in vivo, fructose can be easily used as a glucose substitute by LC cells in vivo by upregulating SLC2A5 [38]. This can be explained by the study of Norimichi et al. [39]. Therefore, SLC2A5-mediated fructose utilization in vivo must play an important role in the control of LC growth, especially in LUAD. However, if there is too much fructose in the gut, the unabsorbed fructose may lead to bacterial fermentation, leading to irritable bowel syndrome, resulting in inflammatory damage, flora disorders, bacterial infections, and other harmful consequences, which may affect the progress of tumor [40]. Although LUAD treatment strategies have improved significantly in recent decades, survival rates still remain unsatisfactory. Therefore, it is necessary to develop new prognostic biomarkers and therapeutic targets. In our study, we explored the expression, prognostic role, and biological function of SLC2A5 in LUAD.

Results of our study found that SLC2A5 is highly expressed in LUAD tumor tissues and is also significantly associated with its prognosis. Moreover, each tumor stage also has high expression, at the same time, the correlation between SLC2A5 expression and prognosis based on different clinical characteristics indicates that SLC2A5 may be a potential independent biomarker for LUAD prognosis. Then, we analyzed the correlation between SLC2A5 and immune infiltration and the correlation between SLC2A5 and different subsets of immune cells. And it is also found that SLC2A5 is related to the major infiltrating immune cells and has a particularly strong effect on B cell and dendritic cell infiltration. Therefore, SLC2A5 infiltration in B cells and dendritic cells may be one of the factors of its prognostic ability. And then, we analyzed the effect of SLC2A5 expression on promoter methylation. Finally, we studied the SLC2A5 coexpression and regulatory networks. All the work above we do is aimed at guiding LUAD future research.

It is worth noting that different lymph node metastasis statuses (N classification) are highly correlated with SLC2A5 expression and SLC2A5 high expression occurred more significantly in N3 than in N0, N1, and N2, which suggests that SLC2A5 is mainly involved in the N3 stage of LUAD [17, 41] and that there may be a relationship between SLC2A5 expression and LUAD disease outcomes, that is to say, the overexpression of SLC2A5 enhances the proliferation, migration, invasion, and tumorigenicity of cells and then closely affects the progress of LUAD [7]. Therefore, we used the PrognoScan database and its related data to carry out survival analysis and meta-analysis and found that high SLC2A5 expression may be associated with poor OS prognosis of LUAD. Additionally, analysis in the Kaplan-Meier plotter showed the correlation between SLC2A5 expression level and the different clinical features of the LUAD, which further demonstrated that SLC2A5 is an independent risk factor of LUAD. Therefore, our results suggest that SLC2A5 upregulation occurs in LUAD and is worthy of further clinical validation as a potential diagnostic and prognostic marker.

Our study found that SLC2A5 expression level was closely related to B cell and dendritic cell infiltration. Subsequently, Kaplan-Meier analysis found certain correlation between B cells and LUAD prognosis and between dendritic cells and LUAD prognosis. These findings suggest that B cell and dendritic cell infiltration may be key factors in SLC2A5 with prognostic value. Next, in the correlation analysis between SLC2A5 and several immune characteristics, we found that most of the marker genes in immune-infiltrating cells were also associated with high SLC2A5 expression. As the most important prognostic factors of SLC2A5, the gene markers of B cells and dendritic cells were associated with SLC2A5 (correlation between these genes and SLC2A5: *P* < 0.0001). It is worth noting that all the marker genes of natural killer cell (KIR2DL1, KIR2DL3, KIR2DL4, KIR3DL1, KIR3DL2, KIR3DL3, and KIR2DS4) are correlated with the high expression of SLC2A5. These marker genes may affect the occurrence and development of LUAD by regulating the activity and effector function of NK cells. In conclusion, this analysis provides a detailed description of the relationship between SLC2A5 and immune characteristics in LUAD patients, indicating that SLC2A5 is a key factor of immune escape in the tumor microenvironment. In addition, the correlation between SLC2A5 and B cells and dendritic cells and their related markers is also very important for the prognosis of LUAD patients. For example, as the literature [42] shows, SLC2A5 could inhibit the development of human normal adjacent lung adenocarcinoma cytoplasmic pre-B cells. The network mechanism includes Golgi apparatus of AP1M2_1; cell cycle of CUL7, SAC3D1; protein amino acid dephosphorylation of STYXL1; pro-B cell–cell differentiation of SOX4_3; and FAD biosynthesis of FLAD1. Therefore, the correlation between SLC2A5 and B cells and their related markers is very important for the prognosis of LUAD patients. At the same time, it is worth noting that SLC2A5 may be a key factor in mediating dendritic cell therapy, which needs further study.

To investigate the reasons for the increase of SLC2A5 in LUAD, we studied the methylation level of SLC2A5 in the LUAD and found that the methylation level of promoters in the LUAD decreased. Therefore, SLC2A5 may be activated and upregulated by its hypomethylation, which may explain a certain degree elevation of SLC2A5 in LUAD. Among the results of gene enrichment analysis, we found that the biological processes of SLC2A5 and its related genes mainly belong to the immune response of the body, such as adaptive immune response, lymphocyte activation, leukocyte activation, differentiation and migration, and other processes related to immunity. Therefore, SLC2A5 may play a vital role in the immune microenvironment of LUAD by participating in the immune response and possibly regulating immune cells such as B cells and T cells and interferon *γ*, interleukin-10, interleukin-12, and other immune regulatory factors. Last but not least, in the construction of the gene coexpression network, we can see that the expression of IL4I1, FCGR2B, and GPR84 has the strongest positive correlation with the expression of SLC2A5, while the expression of TOB1, SELENBP1, and IL17RE has the strongest negative correlation with the expression of gene coexpression. According to some relevant paper, the role of IL4I1 in escaping tumor immune response can be achieved by participating in the fine control of adaptive immune of B and T cells [43]. The mouse model of CD8T cell-FCGR2B deletion established by Anna et al. demonstrated the intrinsic coinhibitory function of Fc*γ*RIIB (FCGR2B) in regulating the immunity of CD8 T cells [44]. As a member of the metabolic G protein-coupled receptor family, results of Recio et al. have shown that when the body is in an inflammatory state, GPR84 could act as an enhancer of inflammatory signals in macrophages, resulting in increased expression of key inflammatory cytokines and chemokines [45]. The evidence above may explain that SLC2A5 and its related genes are mainly enriched in KEGG pathways related to inflammatory response such as NF-kappa B signaling pathway and Toll receptor signaling pathway. With regard to genes negatively associated with SLC2A5, a previous study showed that overexpression of TOB1 significantly inhibited the proliferation and metastasis of lung cancer cells [46]. TOB1 could inhibit the proliferation and metastasis of lung cancer cells through a series of downstream regulators, including cyclin D1, AKT signal transduction, BCL-2, BCL-XL, and SMAD4 [47]. SELENBP1 is a direct target for transcription factor Nkx2-1, which can inhibit tumor clonal growth and migration and inhibit malignant progression of LUAD in vivo. Thus, SELENBP1 is an important inhibitor of lung tumor growth and plays a role in the positive feedback loop of Nkx2-1 [48]. The loss of the expression of this gene may be associated with poor prognosis in lung cancer patients. As a marker cytokine for TH17 cell subsets [49], IL17RE may help to reshape tumor microenvironment and tumor growth/survival [50]. These evidences prove that when the expression of SLC2A5 increases, the body may regulate some biological processes by affecting the expression of positive and negative related genes, thus affecting the condition of patients with lung adenocarcinoma. However, in this study, all the analyses in this paper are based on servers or databases, which may be different in specific experiments. It will be important to verify the analysis results through experiments in our future research.

## 5. Conclusions

In conclusion, this study provides comprehensive evidence for the value of SLC2A5 in lung cancer progression and its potential as a biological target and prognostic predictor of LUAD. Our findings suggest that the upregulation of SLC2A5 in LUAD predicts adverse outcomes in the overall survival of patients, possibly due to multiple physiological processes affecting SLC2A5 expression. Furthermore, we found a significant correlation between SLC2A5 and most immune features. Nevertheless, attention needs to be paid to the link among SLC2A5 and B cells and dendritic cells and their associated markers, which may be a new direction for future LUAD research.

## Figures and Tables

**Table 1:** Correlation of SLC2A5 mRNA expression and clinical prognosis in LUAD with different important clinical characteristics by Kaplan-Meier plotter.

Factor	Overall survival (*n* = 719)			Progression-free survival (*n* = 461)		
	*N*	HR	*P* value	*N*	HR	*P* value
Sex						
Female	317	1.15 (0.79-1.68)	0.4656	235	1.35 (0.85-2.12)	0.199
Male	344	1.46 (1.05-2.03)	0.0232	226	1.94 (1.25-3.03)	0.0028
Stage						
1	370	1.62 (1.09-2.39)	0.0156	283	2.33 (1.41-3.86)	0.0007
2	136	0.75 (0.46-1.22)	0.2407	103	1.15 (0.67-2)	0.6103
3	24	1.46 (0.54-3.95)	0.4495	10	—	—
4	4	—	—	0	—	—
AJCC stage T						
1	123	2.06 (1.11-3.83)	0.0188	47	1.37 (0.31-6.11)	0.6808
2	105	0.93 (0.54-1.6)	0.7933	93	0.86 (0.46-1.62)	0.6515
3	4	—	—	2	—	—
4	0	—	—	0	—	—
AJCC stage N						
0	184	1.5 (0.93-2.42)	0.097	102	1.63 (0.75-3.55)	0.2142
1	44	0.73 (0.34-1.6)	0.4358	38	1.06 (0.43-2.62)	0.8979
2	3	—	—	2	—	—
AJCC stage M						
0	231	1.55 (1.04-2.31)	0.0315	142	1.77 (0.99-3.18)	0.0514
1	1	—	—	0	—	—
Smoking history						
Exclude those never smoked	246	2.68 (1.62-4.44)	6.70*E* − 05	243	2.05 (1.31-3.2)	0.0013
Only those never smoked	143	1.77 (0.78-4.06)	0.1686	143	2.32 (1.22-4.41)	0.0081

Bold values indicate *P* < 0.05.

**Table 2:** Correlation analysis between SLC2A5 and related genes and markers of immune cells in TIMER.

Description	Gene markers	LUAD			
		None		Purity	
		Cor	*P* value	Cor	*P* value
CD8+ T cell	CD8A	0.403	0	0.286	∗∗∗
	CD8B	0.343	∗∗∗	0.249	∗∗∗
T cell	CD3D	0.402	∗∗∗	0.279	∗∗∗
	CD3E	0.41	∗∗∗	0.28	∗∗∗
	CD2	0.408	∗∗∗	0.283	∗∗∗
B cell	CD19	0.391	∗∗∗	0.279	∗∗∗
	CD79A	0.45	0	0.351	∗∗∗
Monocyte	CD86	0.599	0	0.538	0
	CSF1R	0.525	∗∗∗	0.456	∗∗∗
TAM	CCL2	0.427	0	0.361	∗∗∗
	CD68	0.469	0	0.398	∗∗∗
	IL10	0.424	∗∗∗	0.346	∗∗∗
M1 macrophage	NOS2	0.153	∗∗	0.085	5.89*E* − 02
	IRF5	0.36	∗∗∗	0.287	∗∗∗
	PTGS2	0.075	8.85*E* − 02	0.081	7.35*E* − 02
M2 macrophage	CD163	0.517	0	0.458	∗∗∗
	VSIG4	0.435	0	0.375	∗∗∗
	MS4A4A	0.438	0	0.368	∗∗∗
Neutrophils	CEACAM8	-0.134	∗	-0.158	∗∗
	ITGAM	0.479	0	0.414	∗∗∗
	CCR7	0.255	∗∗∗	0.113	1.23*E* − 02
Natural killer cell	KIR2DL1	0.189	∗∗∗	0.143	∗
	KIR2DL3	0.256	∗∗∗	0.187	∗∗∗
	KIR2DL4	0.464	∗∗∗	0.41	∗∗∗
	KIR3DL1	0.178	∗∗∗	0.129	∗
	KIR3DL2	0.288	∗∗∗	0.23	∗∗∗
	KIR3DL3	0.245	∗∗∗	0.241	∗∗∗
	KIR2DS4	0.253	∗∗∗	0.201	∗∗∗
Dendritic cell	HLA-DPB1	0.209	∗∗∗	0.084	6.35*E* − 02
	HLA-DQB1	0.189	∗∗∗	0.081	7.33*E* − 02
	HLA-DRA	0.252	∗∗∗	0.139	∗
	HLA-DPA1	0.226	∗∗∗	0.109	1.52*E* − 02
	CD1C	-0.062	1.57*E* − 01	-0.166	∗∗
	NRP1	0.187	∗∗∗	0.163	∗∗
	ITGAX	0.561	∗∗∗	0.482	∗∗∗
Th1	TBX21	0.366	∗∗∗	0.247	∗∗∗
	STAT4	0.307	∗∗∗	0.184	∗∗∗
	STAT1	0.468	0	0.397	∗∗∗
	IFNG	0.443	∗∗∗	0.361	∗∗∗
	TNF	0.303	∗∗∗	0.207	∗∗∗
Th2	GATA3	0.325	∗∗∗	0.213	∗∗∗
	STAT6	-0.138	∗	-0.135	∗
	STAT5A	0.39	∗∗∗	0.291	∗∗∗
	IL13	0.057	1.99*E* − 01	-0.019	6.71*E* − 01
Tfh	BCL6	0.098	2.65*E* − 02	0.092	4.14*E* − 02
	IL21	0.361	∗∗∗	0.314	∗∗∗
Th17	STAT3	0.104	1.80*E* − 02	0.115	1.07*E* − 02
	IL17A	0.201	∗∗∗	0.13	∗
Treg	FOXP3	0.48	0	0.389	∗∗∗
	CCR8	0.434	∗∗∗	0.351	∗∗∗
	STAT5B	0.209	∗∗∗	0.196	∗∗∗
	TGFB1	0.308	∗∗∗	0.219	∗∗∗
T cell exhaustion	PDCD1	0.508	∗∗∗	0.415	∗∗∗
	CTLA4	0.485	0	0.388	∗∗∗
	LAG3	0.499	0	0.413	∗∗∗
	HAVCR2	0.572	0	0.507	∗∗∗
	GZMB	0.567	∗∗∗	0.491	∗∗∗

TAM: tumor-associated macrophage; Th: T helper cell; Tfh: follicular helper T cell; Treg: regulatory T cell; Cor: *R* value of Spearman's correlation; none: correlation without adjustment; purity: correlation adjusted by purity. ^∗^*P* < 0.01; ^∗∗^*P* < 0.001; ^∗∗∗^*P* < 0.0001.

## Data Availability

The data that support the findings of this study are available from the corresponding author upon reasonable request.
